# Comprehensive characterization of the structure of Zr-based metallic glasses

**DOI:** 10.1038/s41598-024-53509-y

**Published:** 2024-02-28

**Authors:** Debdutta Lahiri, K. V. Mani Krishna, Ashok K. Verma, P. Modak, B. Vishwanadh, Soma Chattopadhyay, Tomohiro Shibata, S. K. Sharma, Sudip Kumar Sarkar, Peter H. Clifton, A. Biswas, Nandini Garg, G. K.Dey

**Affiliations:** 1 High Pressure and Synchrotron Radiation Physics Division, Bhabha Atomic Research Centre, Mumbai, 400085 India; 2 Materials Science Division, Bhabha Atomic Research Centre, Mumbai, 400085 India; 3 Physical Sciences Department, Elgin Community College, 1700 Spartan Drive, Elgin, IL 60123 USA; 4 Materials Science, Kennametal Inc., 1600 Technology Way, Latrobe, PA 15650 USA; 5 Radiochemistry Division, Bhabha Atomic Research Centre, Mumbai, 400085 India; 6 AMETEK Inc., 1100 Cassatt Road, Berwyn, PA USA; 7 Materials Group, Bhabha Atomic Research Centre, Mumbai, 400085 India

**Keywords:** Materials science, Physics

## Abstract

Structure of metallic glasses fascinates as the generic amorphous structural template for ubiquitous systems. Its specification necessitates determination of the complete hierarchical structure, starting from short-range-order (SRO) → medium-range-order (MRO) → bulk structure and free volume (FV) distribution. This link has largely remained elusive since previous investigations adopted one-technique-at-a-time approach, focusing on limited aspects of any one domain. Reconstruction of structure from experimental data inversion is non-unique for many of these techniques. As a result, complete and precise structural understanding of glass has not emerged yet. In this work, we demonstrate the first experimental pathway for reconstruction of the integrated structure, for ${\text{Zr}}_{{{67}}} {\text{Ni}}_{{{33}}}$ and ${\text{Zr}}_{{{52}}} {\text{Ti}}_{{6}} {\text{Al}}_{{{10}}} {\text{Cu}}_{{{18}}} {\text{Ni}}_{{{14}}}$ glasses. Our strategy engages diverse (× 7) multi-scale techniques [XAFS, 3D-APT, ABED/NBED, FEM, XRD, PAS, FHREM] on the *same* glass. This strategy complemented mutual limitations of techniques and corroborated common parameters to generate complete, self-consistent and precise parameters. Further, MRO domain size and inter-void separation were correlated to identify the presence of FV at MRO boundaries. This enabled the first experimental reconstruction of hierarchical subset: SRO → MRO → FV → bulk structure. The first ever image of intermediate region between MRO domains emerged from this link. We clarify that determination of all subsets is not our objective; the essence and novelty of this work lies in directing the pathway towards finite solution, in the most logical and unambiguous way.

## Introduction

Structure of bulk metallic glasses (BMG)^1,2^, has fascinated scientists for (i) its rich physics, manifesting the interplay of geometric, electronic, thermodynamic and kinetic degrees of freedom; (ii) holding the key to glass-forming-ability (GFA)^3,4^ (or conversely, the science of crystallization) and its unprecedented technological promise^5–9^; (iii) representing the generic structural template for ubiquitous systems ranging from electronic devices^10,11^, catalysts^12,13^, engineering materials^6^, liquids^14^, biological systems^15,16^. Therefore, the importance of structural characterization of glass can never be over-emphasized. However, determination of amorphous structure is intrinsically non-trivial, since it lacks the simplicity of crystalline structure:

- The degree of order varies over length-scales and may be disentangled into domains: (a) atomic clusters as basic motifs or short-range-order (SRO)^17–22^; (b) packing of these clusters into networks or medium-range-order (MRO)^23–31^ and (c) distribution of free volume (FV)^32–37^. These can be integrated to construct a hierarchy of decreasing order: [SRO → MRO → FV → bulk structure]. Since no *one* probe is sensitive to varying order of the different domains or all parameters of the same domain, complete structural characterization of glass would necessitate the exercise of diverse multi-scale probes.
- Amorphous structure is inherently degenerate i.e. structural solution is not unique but distributed over (predictably) “finite” range of values^38–41^. Most techniques are equipped to determine the “average” structure and not the “variance”. As a result, reconstruction of glass structure from experimental data inversion is almost always non-unique^42^. Robust strategy is required to resolve the correct solution range.

Properties of glass are contributed by (SRO, MRO, FV) domains as well as inter-domain connection. Therefore, the structural correlation has to be understood in entirety, encompassing all aspects of all domains. This essentially implies that determination of comprehensive structure is indispensable (not redundant) for glass. Further, degeneracy of structural solution has to be adequately resolved to ascertain the correlation unambiguously. The net desired outcome is *complete* and *precise* structural solution, within the practical limitations (described above).

Diverse multi-scale structural probes have been devised. In SRO domain, site-resolved average cluster parameters are determined with “X-ray Absorption Fine Structure” (XAFS)^20–22,43^ (or its combination with XRD^44–46^); *individual* atomic positions are imaged with “Atom Probe Tomography” (3D-APT)^47–50^ and “Atomic Electron Tomography” (AET)^51–53^, atomic arrangement within *individual* clusters are determined with “Angstrom Beam Electron Diffraction” (ABED)^54^. In MRO domain, conventional diffraction has limited success due to decreased order^55–57^. MRO domain size can be determined with “Fluctuation Electron Microscopy” (FEM)^58,62^; *individual* networks are detected with “Nano Beam Electron Diffraction” (NBED)^63^. Free volume (FV) is the least quantified domain, except for estimate of void content with “Positron Annihilation Spectroscopy” (PAS)^64–66^ and spatial distribution of FV with “Filtered High Resolution Electron Microscopy” (FHREM)^67^. History of experiments on metallic glass largely represents *one-technique-at-a-time* approach (probably justified against the complexities involved), delivering segregated pieces of information for *separate* glasses. Even this bit of information is incomplete and uncertain, due to the intrinsic limitations of techniques (described earlier). Thus, the desired outcome of *complete* and *precise* structure did not emerge for any metallic glass so far.

The challenge henceforth is experimental reconstruction of the complete structure, to the best possible extent and precision. Algorithm for the same has not been proposed yet, which is the gap our present work attempts to bridge. We demonstrate *multi*-technique based pathway to achieve this goal, by correcting upon the shortcomings of *individual* techniques:

- The strategy primarily exercises a *combination* of 7 techniques to probe (SRO, MRO, FV) domains of the *same* glass. The strategy works by (i) eliminating artefact from sample-to-sample variation; (ii) mutually complementing limitations of individual techniques, to cover all aspects of each domain; (iii) corroborating common results from different techniques, to generate self-consistent and precise parameters; (iv) covering all domains of the *same* glass, to enable unambiguous derivation of inter-domain link [SRO → MRO → FV → bulk structure].
- First-principles-molecular-dynamics (FPMD) modeling is integral to our strategy, in order to accommodate both model-independent and model-based fitting of experimental data. If the results from the two fitting strategies agree for all techniques, such concomitant validation will bring reasonable certainty to FPMD model, in the most unbiased way. This will narrow down the degeneracy of structural solution toward “finite” FPMD model.

The whole (above) exercise is repeated for multiple glasses with vastly different GFA, in order to test if structural results are commensurate with relative GFA. This is crucial in order to reinforce that the results are not ad-hoc but scientifically founded. We clarify that our objective is not to reconstruct all individual clusters or their connection (which is impractical) but to direct the experimental pathway towards finite and comprehensive solution for any glass, which has never been considered before.

In this work, we undertake this challenge for binary $(B)$ $Zr_{67} Ni_{33}$ *a*nd multi-component $(MC)$ $Zr_{52}\, Ti_{6}\, Al_{10}\, Cu_{18} \,Ni_{14}$^68^ glasses that have vastly different GFA $(B < < MC)$. [$MC$-glass could be obtained in BMG form but not $B$-glass.] We employed a spectrum of multi-scale experimental techniques (XRD, XAFS, FEM, 3D-APT, ABED/NBED, PAS, FHREM), listed in Table 1. [Their brief description is presented in supplementary material (S1.doc).] Reference FPMD model was generated for $250 - 686$-atom ensembles^58^, quenched at 4–12$\times 10^{13} \quad {\text{K/s}}$. FPMD results are robust $( \pm 2\% )$ relative to ensemble size, due to the exercise of exact potentials without explicit assumption (unlike classical MD). Cooling rate-dependent structural variations are similar to those observed by other researchers^69,70^.

**Table 1:** List of techniques employed for structural study of glasses.

Techniques	Probe size	Results
X-ray absorption fine structure (XAFS)	mm	Average cluster information around a particular elemental species: composition, bond-lengths and distortion
3D—atom probe tomography (APT)	> 10 nm	Spatial distribution of elements Cluster coordination and composition distribution
Angstrom beam electron diffraction (ABED)	0.5 nm	symmetry of atomic arrangement in individual clusters
Nanobeam beam electron diffraction (NBED)	1.0 nm	Ordering of clusters
Fluctuation electron microscopy (FEM)	1.43–6.77 nm	MRO domain size; Atomic arrangements over MRO scale
X-ray diffraction	mm	Ensemble-averaged radial distances between nearest neighbors and second nearest neighbors
Positron annihilation spectroscopy (PAS)	mm	Void size and frequency
Filtered high resolution electron microscopy (FHREM)	0.4 nm	Void size and spatial distribution

 FPMD model reveals the existence of broad but “finite” and non-random ensemble, defined by structural and chemical constraints: (a) SRO: element-resolved cluster parameters [coordination, symmetry, size and distortion] and (b) MRO: SRO-specific cluster-pairing, mode of cluster-connection and size of network. We validated FPMD parameters of (SRO, MRO) domains with 2–3 techniques each, with model-independent and (FPMD) model-based fitting. Concomitant validation by multiple techniques brought reasonable certainty to the FPMD model. This enabled narrowing down of structural degeneracy toward FPMD model, with greater confidence than in previous works. In particular, we exploited the high resolution of *local* techniques (ABED/ NBED) to precisely decipher 1–2 individual FPMD clusters and $1$ FPMD MRO domain of icosahedral (ISRO) clusters extending over ${1}{{.2}}\;{\text{nm}}$. Reproduction of ABED/NBED patterns with *selected* clusters/networks (out of $500$-atom FPMD ensemble) warrants the solution to be reasonably unique. In FV domain, voids content and inter-void separation were determined with PAS and FHREM respectively. The definition of FV necessitates that chain of atoms will be discontinued at FV. Thus, the presence of FV in a region unambiguously signifies that one MRO domain has terminated there and another MRO domain will commence from there. We present the first experimental evidence towards this, by demonstrating that inter-FV separation (from FHREM) equals MRO domain size ($\Lambda$) in both the glasses. This essentially implies that consecutive FV are separated by intermediate MRO domain of size ($\Lambda$). Or conversely, FV is located at the boundaries of this MRO domain, thereby justifying the definition of FV. This novel finding unravels the intermediate region of low atomic density between MRO domains for the first time. Cumulatively, this accomplished experimental reconstruction of *one* hierarchical subset [SRO → MRO → FV → bulk structure] of the whole structure, with significant *precision*. The solution is also warranted to be scientifically robust, as the relative parameters of the two glasses are commensurate with their relative GFA.

We finally comment on the merit of our strategy vis-à-vis direct imaging methods (APT/ AET) of 3D-atomic positions^47–53^. Limitations of these direct probes have been addressed in Ref. 52, out of which we particularly emphasize on uncertainty (± 0.2–2 Å) in the determination of atomic positions. Since shifts in atomic positions of this scale can alter bond energy, cluster geometry and cluster packing appreciably, comparison of experimental results with any theoretical model becomes meaningless. Our strategy included 3D-APT for precise cluster composition information while spatial information (± 0.01 Å) was supplemented with XAFS. Further, FV distribution is not clearly evidenced from APT/AET probes, due to poor spatial resolution. Cumulatively, multi-technique approach generates far more comprehensive and precise information, vis-à-vis direct imaging.

## Results

### Short-range-order (SRO)

#### First principle molecular dynamics (FPMD) calculations

**Figure 1:**
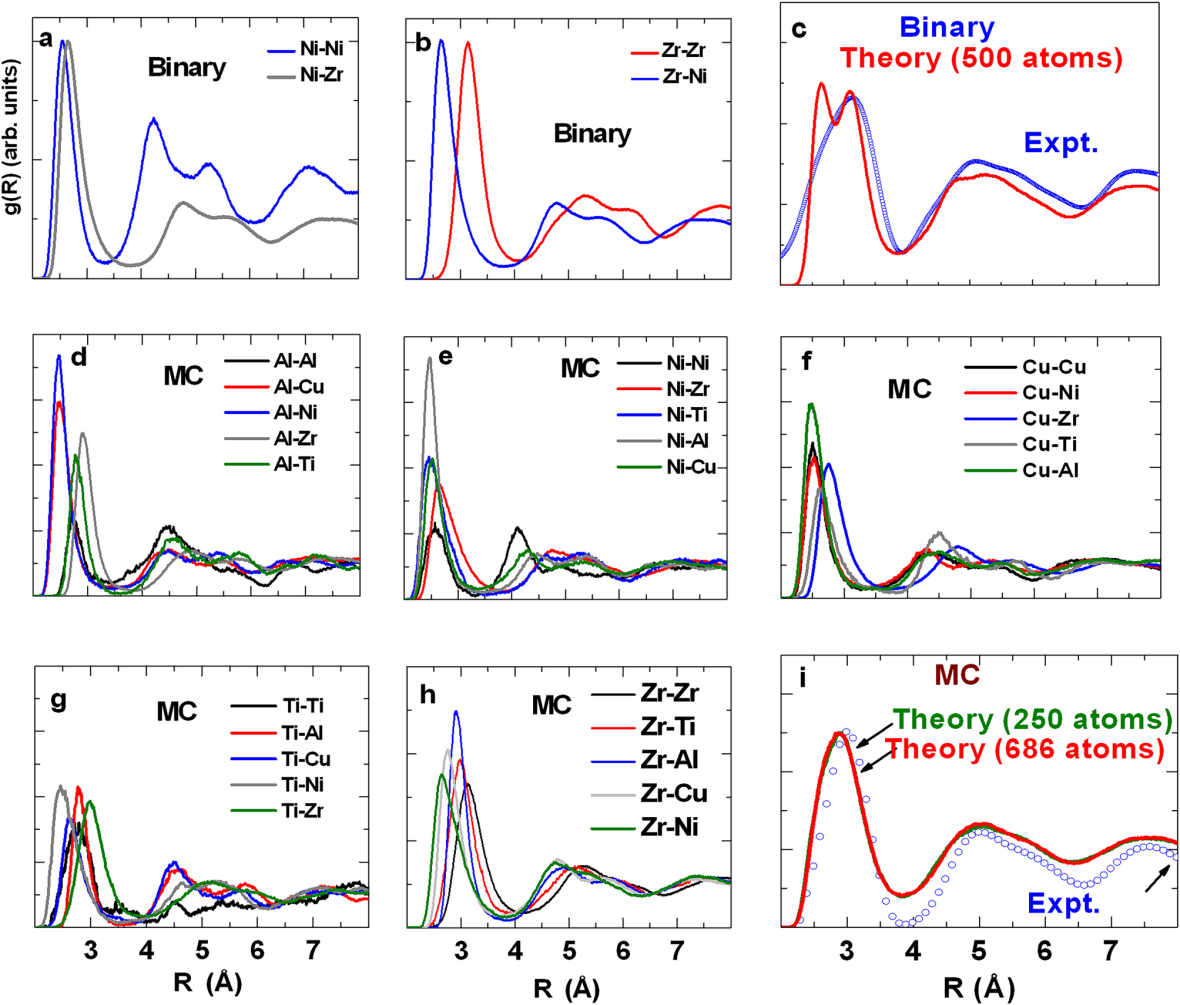
Partial pair distribution for (**a,b**) binary (B) and (**d–h**) multi-component (MC) glasses. Total radial distribution function (*rdf*) for (**c**) binary (B) and (**i**) multi-component (MC) glasses. In (**c,i**), experimental *rdf* derived from XRD are over-plotted.

FPMD results are presented for $(500,\;250)$-atom ensemble sizes for ${\text{Zr}}_{{{67}}} {\text{Ni}}_{{{33}}}$(B) and ${\text{Zr}}_{{{52}}}\, {\text{Ti}}_{{6}}\, {\text{Al}}_{{{10}}}\, {\text{Cu}}_{{{18}}} \, {\text{Ni}}_{{{14}}}$(MC) respectively (for MC-glass, calculations were repeated for $686$-atom ensemble size to confirm robustness of results). SRO is characterized for arrangement of nearest neighbor atoms around each site, which we define as cluster. Ensemble-averaged partial radial distribution functions (${\text{g}}_{{{\text{ij}}}} {\text{(R)}}$) for $Zr_{67}\, Ni_{33}$ and $Zr_{52}\, Ti_{6}\, Al_{10} \,Cu_{18} \,Ni_{14}$ are shown in Fig. 1a–b and d–h respectively, where the first maxima represents nearest neighbor correlation. Theoretical total radial distribution function [$g(r)$] were generated for $500$-atom (B) and [${250}$,${686}$]$(250 \, \mathrm{ and }\, 686)$-atom (MC) ensembles (Fig. 1c, i), by integrating partial pair distribution functions of Fig. 1a, b and d–h respectively. Negligible variation of $g(r)$ between [${250}$,${686}$]$(250\, \mathrm{ and }\, 686)$-atom ensemble sizes (Fig. 1i) confirms that FPMD results are robust relative to ensemble size. First maxima positions of $g(r)$ (around 0.28 nm) correspond to average nearest neighbor distance for each glass. Cluster parameters of each ensemble are derived from these distributions.

### (a) Cluster coordination

**Figure 2:**
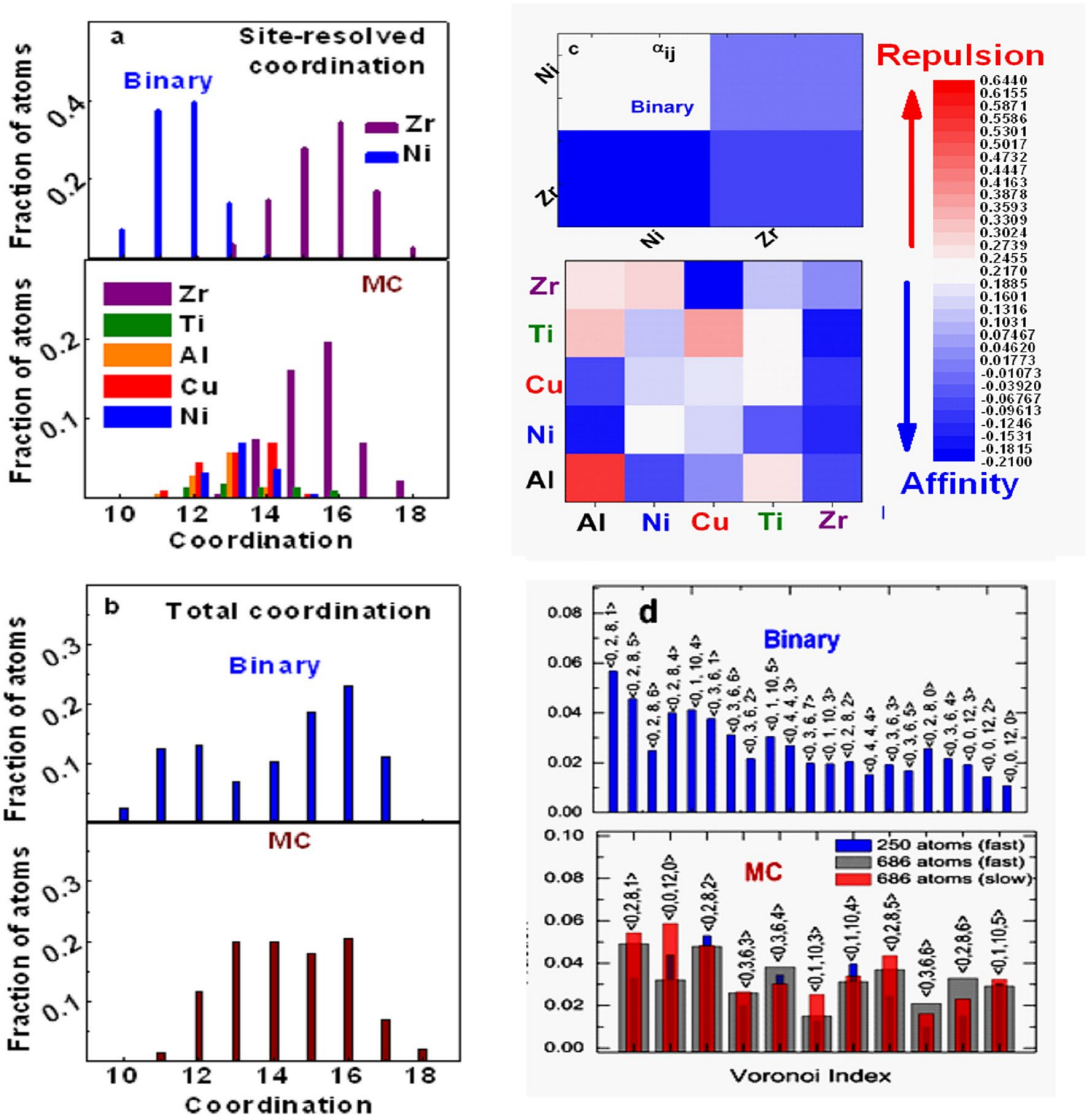
For binary (B) and multi-component (MC) glasses: (**a**) site-resolved and (**b**) total coordination distributions of FPMD ensemble; (**c**) chemical interaction matrix for atomic pairs; (d) Voronoi polyhedral distribution (for MC-glass, polyhedral distributions are shown for two different ensemble sizes and cooling rates).

For each glass, partial pair coordination ${\text{N}}_{{{\text{ij}}}}$(Table 2) was extracted by integrating each ${\text{g}}_{{{\text{ij}}}} {\text{(R)}}$ of Fig. 1, over the first maxima up to the first minimum. Total cluster coordination around $i{\text{th}}$ element ${\text{(N}}_{{\text{i}}} = \sum\nolimits_{{\text{j}}} {{\text{N}}_{{{\text{ij}}}} } {)}$ conforms to solute–solvent atomic size ratio $(R^{*} )$^71^ e.g. cluster coordination increases for $Ni \to Zr$ centers (Table 2). Average cluster coordination around $(Ni,Zr)$ sites for the two glasses: ${\text{N}}_{{{\text{Ni}}}} \approx {\text{12(B)}} \to {13}({\text{MC)}},{\text{N}}_{{{\text{Zr}}}} \approx {\text{14(B)}} \to {\text{16(MC)}}$. These are site-averaged results; in reality, each element hosts clusters of variable coordination extending over finite range $\Delta N_{i} = 5$ (Fig. 2a). Such a narrow range represents “quasi-equivalent cluster” model. Site-integrated coordination distributions for the whole ensemble are presented in Fig. 2b. These distributions (Fig. 2b) emulate the stoichiometric contrast of the alloys viz. bimodal $(B)$ vis-à-vis broad Gaussian $(MC)$. Bimodal distribution for $B$-glass is peaked around ${\text{N}} = \left[ {{11}{\text{.5(}} \approx {\text{N}}_{{{\text{Ni}}}} {),16(} \approx {\text{N}}_{{{\text{Zr}}}} {)}} \right]$, corresponding to $(Ni,Zr)$-sites. On the other hand, uniform distribution (${\text{(N}} = {13 - 16)}$ for $MC$-glass covers larger range of sites ${\text{(Al, Ti, Ni, Cu, Zr)}}$.

**Table 2:** Fractional Pair Coordination from FPMD model and APT analysis.

Fractional pair coordination	Binary	Binary	MC	MC
	FPMD	APT	FPMD	APT
Al-centre				
AlAl	–	–	0.04677	0.07689
AlNi	–	–	0.15476	0.10848
AlCu	–	–	0.18027	0.18524
AlTi	–	–	0.04592	0.06269
AlZr	–	–	0.57228	0.5667
Cu-centre				
CuAl	–	–	0.10679	0.07694
CuNi	–	–	0.11765	0.10718
CuCu	–	–	0.15023	0.18646
CuTi	–	–	0.04615	0.06367
CuZr	–	–	0.57919	0.56576
Ni-centre				
NiAl	–	–	0.12152	0.07801
NiNi	0.17925	0.33	0.05102	0.10894
NiCu	–	–	0.15955	0.18543
NiTi	–	–	0.06494	0.06312
NiZr	0.82075	0.67	0.60297	0.5645
Ti-centre				
TiAl	–	–	0.07052	0.07675
TiNi	–	–	0.12758	0.10758
TiCu	–	–	0.12044	0.18755
TiTi	–	–	0.04754	0.06611
TiZr	–	–	0.63391	0.56202
Zr-centre				
ZrAl	–	–	0.07924	0.07757
ZrNi	0.3007	0.32	0.10688	0.10754
ZrCu	–	–	0.22727	0.18649
ZrTi	–	–	0.05651	0.06295
ZrZr	0.6993	0.68	0.5301	0.56545

### (b) Cluster composition

Site-resolved coordination $(N_{i} )$ can be further resolved into partial pair coordination (${\text{N}}_{{{\text{ij}}}}$). Site-resolved fractional pair coordination $\left( { = \frac{{{\text{N}}_{{{\text{ij}}}} }}{{N_{i} }}} \right)$ is listed in Table 2. Dominance of hetero-atomic bonds (e.g. ${\text{Ni Zr}}$) is demonstrated for both glasses. In order to decouple strength of chemical interaction between atomic pairs from stoichiometric abundance $(x_{j} )$, chemical interaction strength $(\alpha_{ij} )$ is quantified by normalizing fractional composition ${\text{(N}}_{{{\text{ij}}}} )$ wrt $x_{j}$^72^: $\alpha_{ij} = {1} - \frac{{N_{ij} }}{{x_{j} N}}$; $$\left( \begin{gathered} \alpha_{ij} = {0} \hfill \\ \alpha_{ij} < {0} \hfill \\ \alpha_{ij} > {0} \hfill \\ \end{gathered} \right)$$ represent random pairing, chemical affinity and repulsion respectively. Relative interaction strengths for different atomic pairs are presented in the matrices of Fig. 2c. The strongest chemical interactions are $Ni - Zr$ and ${\text{[Ni - Al, \,Ni - Zr,\, Cu - Zr,\, Ti - Zr]}}$ for $B$- and $MC$- glasses respectively. The common interaction ($Ni - Zr$) is significantly stronger for $B$-glass; its relative weakening in $MC$-glass could be due to the coexistence of multiple competing interactions. Stronger ${{Ni - Zr}}$ interaction likely enables the formation of ${\text{NiZr}}_{2}$-like SRO in $B$-glass^73^$$\left( \begin{gathered} {\text{N}}_{{{\text{NiNi}}}} \approx {\text{2,N}}_{{{\text{NiZr}}}} \approx {9} \hfill \\ {\text{N}}_{{{\text{ZrNi}}}} \approx {\text{4,N}}_{{{\text{ZrZr}}}} \approx {10} \hfill \\ \end{gathered} \right)_{B}$$ vis-à-vis $MC$-glass $$\left( \begin{gathered} {\text{N}}_{{{\text{NiNi}}}} \approx {0}{\text{.6,N}}_{{{\text{NiZr}}}} \approx {7} \hfill \\ {\text{N}}_{{{\text{ZrNi}}}} \approx {\text{2,N}}_{{{\text{ZrZr}}}} \approx {9} \hfill \\ \end{gathered} \right)_{MC}$$. This demonstrates direct correlation between alloy composition, strength of chemical ordering and SRO.

### (c) Cluster size and distortion

Nearest neighbor bond-lengths ${\text{(R}}_{{{\text{ij}}}} {)}$ are defined by first ${\text{g}}_{{{\text{ij}}}} {\text{(R)}}$ maxima positions in Fig. 1 and listed in Table 3. [Only bond-lengths with XAFS counterparts are shown.] Hetero-atomic bond-lengths are shorter than corresponding sum-of-atomic-radii, consistent with strong chemical interactions. Cluster radius is the weighted average of intra-cluster bond-lengths ${\text{(R}}^{{\text{i}}} = \sum\nolimits_{{\text{j}}} {{\text{f}}_{{{\text{ij}}}} {\text{R}}_{{{\text{ij}}}} } {)}$ and cluster distortion $(\delta^{i} )$ their weighted standard deviation: ${\updelta }^{{\text{i}}} = \sqrt {\sum\nolimits_{{\text{j}}} {{\text{f}}_{{{\text{ij}}}} {\text{(R}}_{{{\text{ij}}}} - {\text{R}}_{{{\text{avg}}}}^{{\text{i}}} {)}^{{2}} } }$. In each glass, both cluster size ${\text{(R}}^{{\text{i}}} {)}$ and distortion $(\delta^{i} )$ increase towards $Zr$-centered clusters due to the formation of longer ${\text{(ZrZr)}}$ bonds. Cluster size around common centers $(Ni,Zr)$ is similar in both glasses. On the other hand, cluster distortion is significantly reduced for $MC$-glass: ${\updelta }^{{{\text{Zr}}}} = {\text{26\% (B)}} \to {\text{14\% (MC}})$, due to the incorporation of multiple elements with intermediate atomic sizes.

**Table 3:** XAFS fit results for bond-length and Debye–Waller factor.

Atomic pair	Sum of atomic radii	Bond-length (Å)		Debye–Waller factor (Å^2^)
		Binary	MC	
		FPMD (XAFS)	FPMD (XAFS)	
Cu-centre				
Cu–Al	2.58	–	2.47 (2.47)	0.004 (MC)
Cu–Cu/Ni	2.55	–	2.55 (2.5)	0.008 (MC)
Cu–Zr	2.81	–	2.76 (2.7)	0.020 (MC)
Ni-centre				
NiNi	2.55	2.63 (–)	–	–
Ni–Zr	2.86	2.64 (–)	2.63 (2.64)	0.010 (MC)
Zr-centre				
Zr–Cu/Ni	2.81, 2.86	2.64 (2.66) NiZr_2_ = 2.76	2.76, 2.63 (2.69)	0.016 (MC) 0.010 (B)
Zr–Zr	3.14	3.14 (3.06) NiZr_2_ = 2.98 3.07	3.14 (3.16)	0.023(MC) 0.025 (B)

### (d) Cluster symmetry

Voronoi polyhedral (VP) analysis reveals the existence of ${\text{[21(B),11(MC)]}}$ primary symmetry groups, distributed over ${\text{[55\% (B),40\% (MC)]}}$ clusters of respective ensembles (Fig. 2d). Remaining ${\text{[45\% (B),60\% (MC}})]$ clusters of each ensemble are distributed over several secondary symmetry groups of $< {\text{1\% }}$ population each. Cumulatively, cluster symmetry distribution is much broader for $MC$-glass. [Total population of all VPs ${(} = {10}0\% )$ fills the space.] ISRO content is significantly higher for $MC$-glass $( = {\text{14\% )}}$ [vis-à-vis $6\% (B)$]^74^, although ${\text{N}} = {12}$ cluster population is comparable for both glasses (Fig. 2b). Low ISRO content of $B$-glass is consistent with its poor GFA and could be the outcome of strong $Ni - Zr$ interaction driving SRO toward crystalline phase (at the cost of ISRO).

[We remark that the above SRO results are derived at cooling rate $\sim 10^{13} K/s$ (vis-à-vis experimental cooling rate $\sim 10^{6} K/s$). Variation due to different cooling rates should be small viz. $\sim 10\%$ for ISRO content and $1 - 10\%$ for other SRO parameters^69,70^. Since these discrepancies are within experimental uncertainties of our techniques, they will practically have no bearing on our broader conclusions].

#### X-ray diffraction

Experimental $g(r)$ were extracted from XRD patterns that reproduce first maxima of FPMD $g(r)$ (Fig. 1c, i). For $B$-glass, split first maxima of FPMD $g(r)$ over ${\text{r}} = {0}{\text{.2}} - {0}{\text{.4 nm}}$ (Fig. 1c) is broadened into single asymmetric experimental peak, due to limited experimental resolution.

#### X-ray absorption fine structure (XAFS)

**Figure 3:**
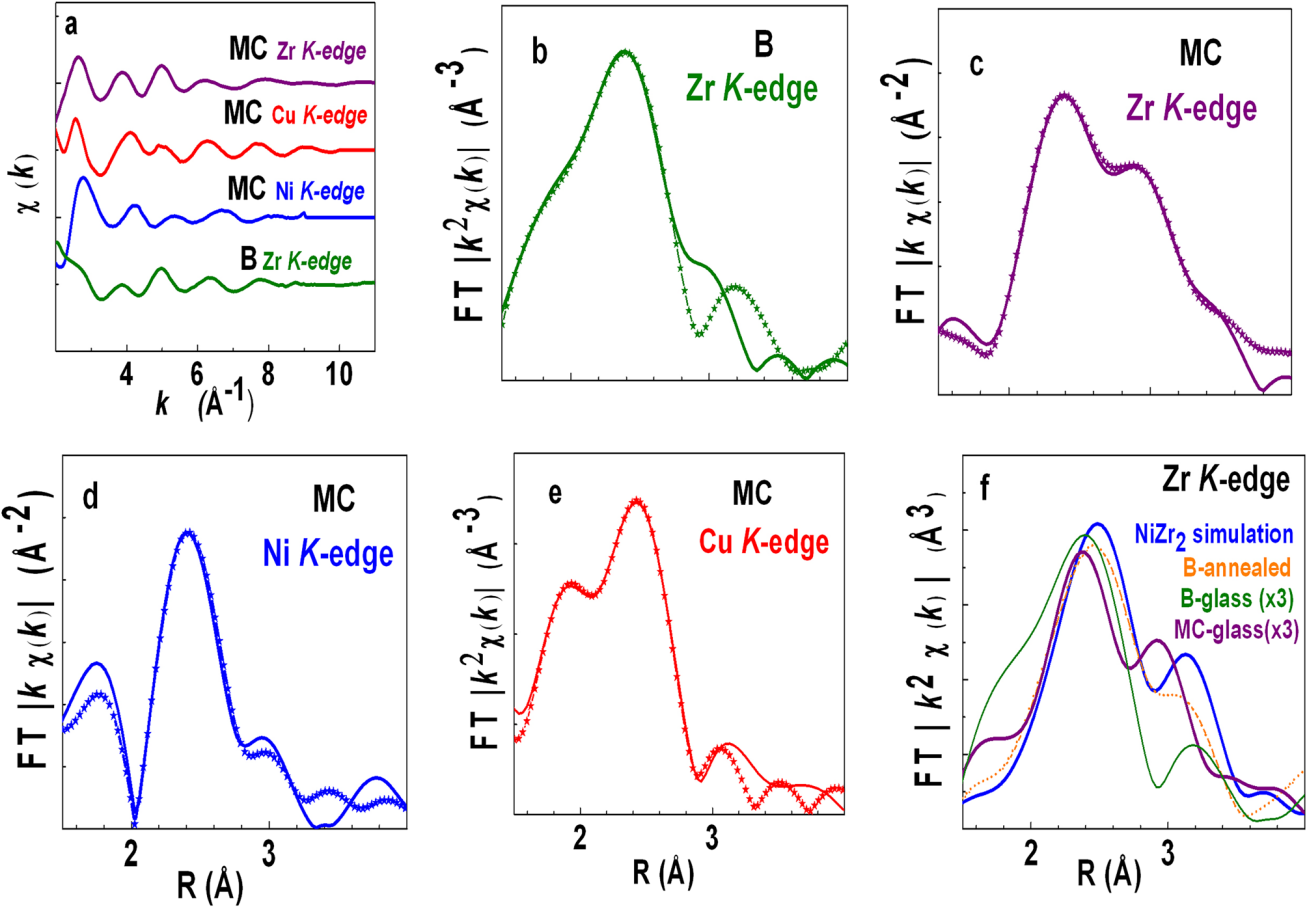
(**a**) XAFS oscillations [*χ*(k)] for binary (B) and MC-glasses. Ni *K*-edge data for binary (B) glass was unstable and therefore, not presented; (**b–e**) comparison of fit with experimental *χ*(R) for (**b**) B-glass and (**c–e**) MC-glass; (**f**) *χ*(R) at Zr *K*-edge for binary alloy (glassy and annealed phases) and MC-glass, compared with simulated *χ*(R) for NiZr_2_. Plots for glassy phases of (B, MC) are multiplied (×3) for clarity.

XAFS data ($\chi (k)$) for $B$- and $MC$-glasses are presented in Fig. 3a—fast decay of XAFS oscillations beyond ${\text{k}} = {\text{10 {\AA}}}^{{{ - }{1}}}$ is typical of amorphous materials (Ni *K*-edge $\chi (k)$ for $B$-glass changed within the course of single scan (< 20 min) as the glass started annealing, subsequent to heat absorption from Ni *K*-edge x-rays. Since this dataset is unsuitable for analysis, it is not presented in Fig. 3a). Fourier transforms [$\chi (R)$] display nearest neighbor peaks over ${1}{\text{.6}} - {3}{\text{.2{\AA}}}$ (Fig. 3b–e) (peak at ${\text{R}} < {1}{\text{.5 {\AA}}}$ is not real but generated by slight oscillatory character of background arising from limited *k-*range. Leakage from background peak is negligible and therefore, XAFS fit results are free of background-related artefact). Both (i) FPMD model-based and (ii) independent fittings were considered.

(i) For independent fitting, bond-length $(R)$, coordination $(N)$ and Debye–Waller factor $(\sigma^{2} )$ were varied for each scattering path. To minimize errors in fit results, common bonds (e.g.${\text{ZrNi}}$) were fit simultaneously at ${\text{(Ni, Zr)}}$ *K*-edges. In the case of $MC$-glass, ${\text{(Ni, Cu)}}$-neighbors were treated equivalent due to indistinguishable backscattering factors and similar bond-lengths; any attempt to separately fit (${\text{ZrNi,}}\;{\text{ZrCu}}$) paths resulted in large error bar. XAFS bond-length results (Table 3) agree with FPMD model within $\pm {0}{\text{.05}}\;{\text{\AA}}$ (atomic pairs of very low XAFS amplitude are not listed in Table 3). Coordination result from independent XAFS fitting is ambiguous for disordered systems^20^ and therefore, not presented.
(ii) FPMD-based fitting procedure is described in our earlier paper^20^. Bond-lengths $\left( R \right)$ and coordination ratio (e.g.$N_{{{\text{CuAl}}}} :N_{{{\text{CuCu}}}} :N_{{{\text{CuZr}}}}$) were constrained from FPMD model. Common coordination multiplicative factor for the paths and Debye–Waller factor $(\sigma^{2} )$ for individual path were varied. Debye–Waller factor $(\sigma^{2} )$ results are listed in Table 3. Good fit quality (Fig. 3b–e) validates cluster parameters of FPMD model.

The contrast of SRO between the two glasses becomes prominent by comparing their ${\text{Zr}}$ $K$-edge [$\chi {\text{(R)}}$] with simulated spectra for tetragonal ${\text{NiZr}}_{2}$ [$\chi_{{{\text{NiZr}}_{{2}} }} {\text{(R)}}$] in Fig. 3f. Peak positions of $\chi {\text{(R)}}$ for $B$-glass resemble $\chi_{{{\text{NiZr}}_{{2}} }} {\text{(R)}}$, suggesting that SRO of $B$-glass is close to crystalline phase (proximity with ${\text{NiZr}}_{2}$ is observed to increase in the annealed phase of $B$-glass, as $\chi {\text{(R)}}$ resembles $\chi_{{{\text{NiZr}}_{{2}} }} {\text{(R)}}$ wrt peak positions and relative amplitudes. This demonstrates tendency of $B$-glass to form crystalline phase). On the other hand,$\chi {\text{(R)}}$ for $MC$-glass is significantly shifted relative to $\chi_{{{\text{NiZr}}_{{2}} }} {\text{(R)}}$, suggesting that SRO of MC-glass is distinct from $NiZr_{2}$.

#### 3D atom probe tomography (3D-APT)

Fractional pair coordination $\left( { = \frac{{{\text{N}}_{{{\text{ij}}}} }}{{\sum {{\text{N}}_{{\text{i}}} } }}} \right)$ was derived from spatial coordinates and elemental identities of atoms of 3D-APT data (Table 2) (absolute values of coordination are not shown, since APT coordination is bound to be underestimated due to inevitable loss of atoms during data acquisition. Even the successfully detected atoms/ions may not be accurately identified due to co-evaporation of ions). Fractional pair coordination trends from APT and FPMD can be considered to be fairly agreeing, within $\pm {\text{5\% }}$.

#### Angstrom beam electron diffraction (ABED)

**Figure 4:**
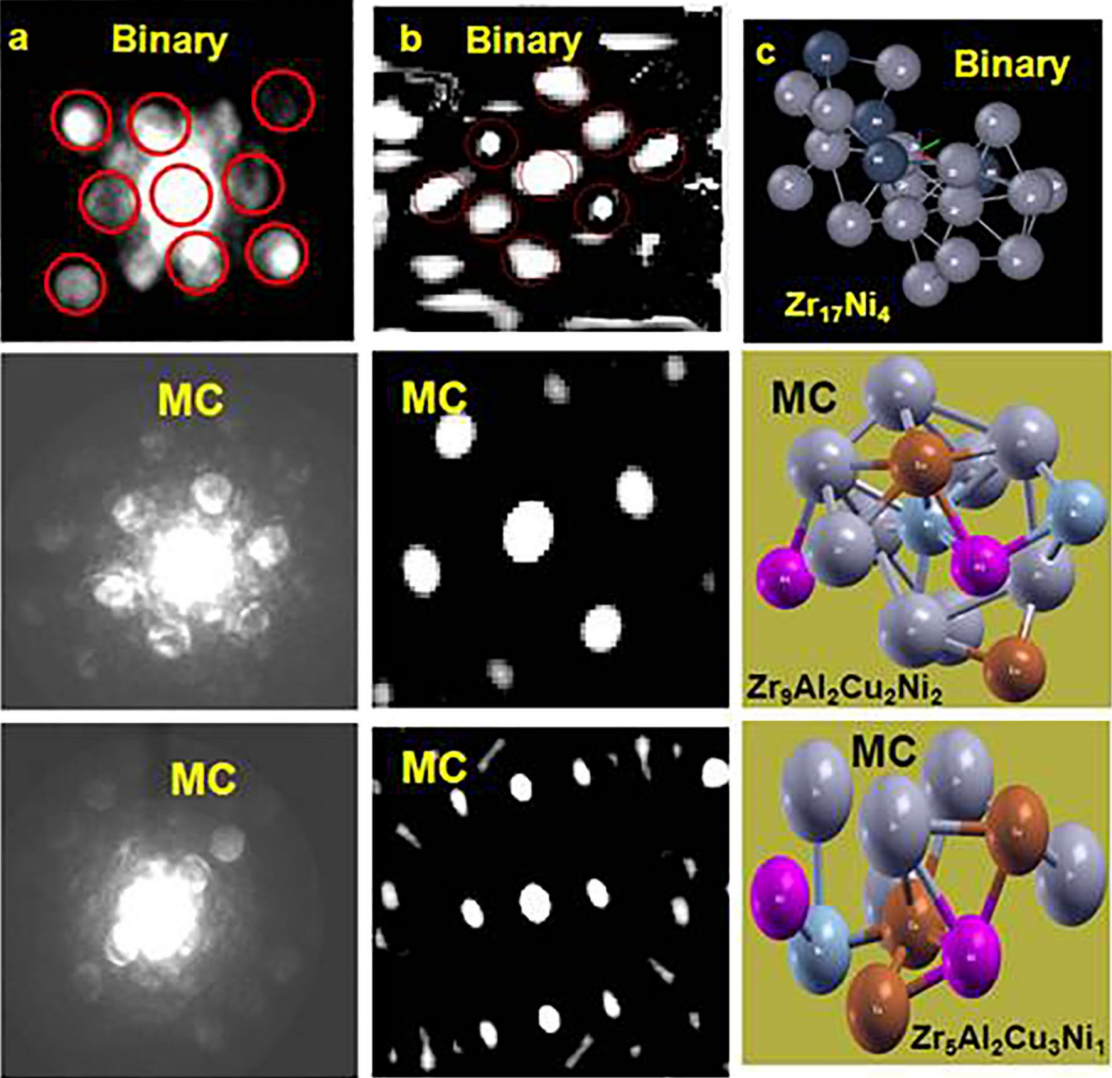
(**a**) Experimental and (**b**) simulated Angstrom beam electron diffraction (ABED) spectra. (**c**) Clusters, corresponding to simulated spectra, are depicted using OVITO Basic v3.7.4 software (Ref. ^83^; https://www.ovito.org/windows-downloads/). Cluster composition is indicated.

Experimental electron diffraction patterns for $[1(B),2(MC)]$ clusters are displayed in Fig. 4a (probability of cluster detection is pre-determined by appropriate cluster orientation for diffraction). Well-defined diffraction spots in the patterns provide direct evidence of local atomic order in both glasses. Since direct reconstruction of atomic positions from these patterns is non-unique, we approached the problem indirectly based on FPMD model. Under this scheme, diffraction patterns were simulated for different clusters of ${(500,686)}$-atom ensembles of $B$- and $MC$-glasses respectively and compared with experimental patterns by trial. The match was evaluated in terms of diffraction spot locations, number and their radial distance from the central (transmitted) spot. The closest-matching simulated patterns and corresponding FPMD clusters are presented in Fig. 4b, c respectively.

### Medium-range-order (MRO)

#### First principles molecular dynamics (FPMD)

**Figure 5:**
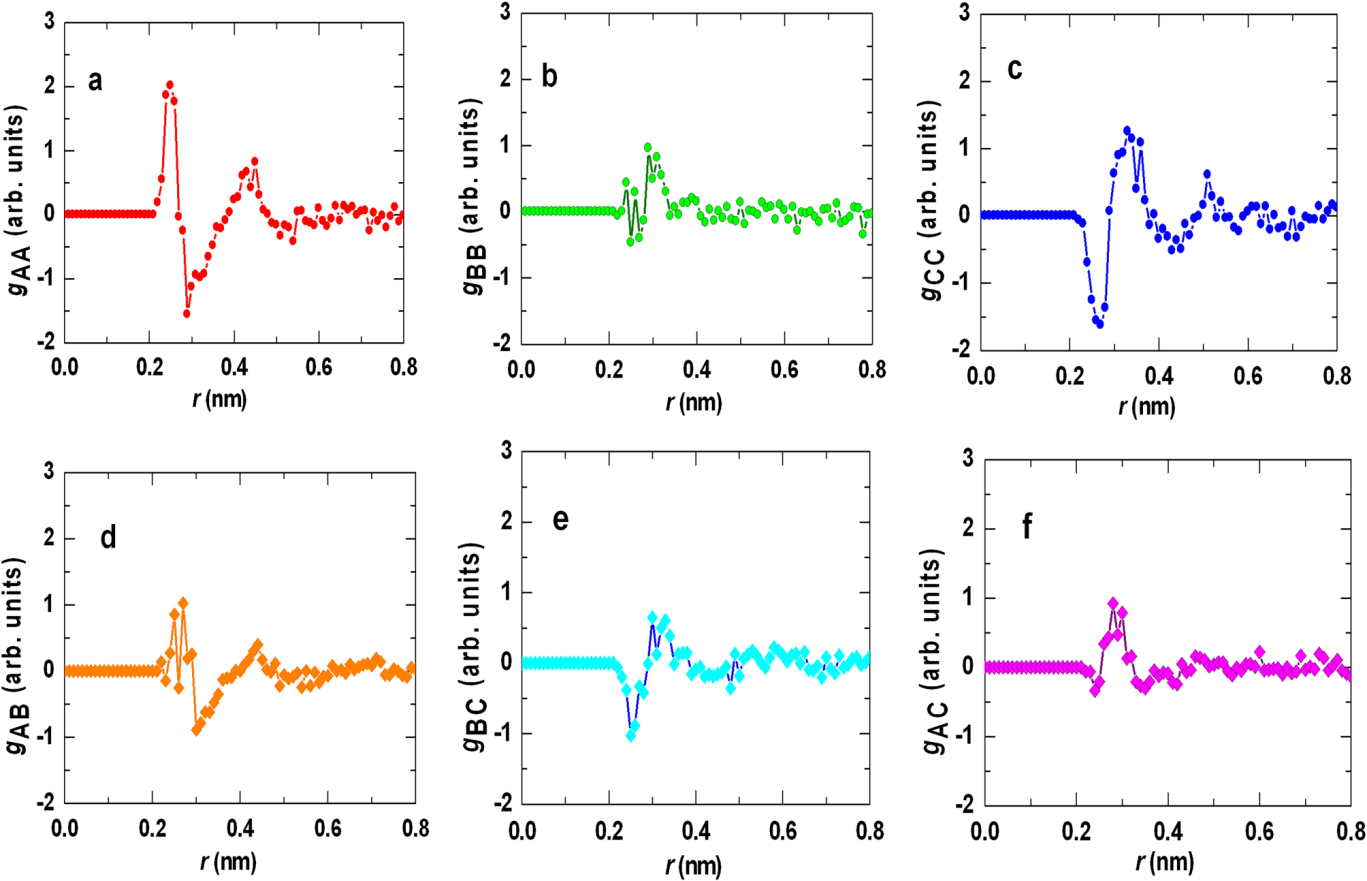
Cluster–cluster pair correlation functions for (A, B, C) = (12, 13, 14)-fold coordinated polyhedra: (**a**) A–A; (**b**) B–B; (**c**) C–C; (**d**) A–B; (**e**) B–C; (**f**) A–C.

MRO was analyzed for MC-glass, on the basis of cluster–cluster correlation ${\text{(G}}_{{{\text{ij}}}} )$ (Fig. 5) for three common cluster groups [${\text{A(N}} = {12)}$ = ISRO, ${\text{B(N}} = {13)}$, ${\text{C(N}} = {14)}$] (this analysis could not be performed for binary glass due to insufficient ISRO cluster content). Clusters were treated as rigid balls located at centre of coordination polyhedron (total pair-correlation function of the glass was subtracted from pair-correlation functions of Fig. 1, which nullified the effect of first order correlations). First (${0}{\text{.2 - 0}}{\text{.35 nm}}$) and second (${0}{\text{.4 - 0}}{\text{.6 nm}}$) maxima of ${\text{G}}_{{{\text{ij}}}}$ represent nearest and second-nearest neighboring cluster-correlations respectively; no subsequent maxima were observed. Therefore, radial distance ${{\sim 0}}{\text{.5 nm}}$ defines the maximum extent of cluster correlation or mean MRO domain size ${{\sim 1}}{\text{.0 nm}}$.

Cluster pairing strengths, derived from amplitudes of first ${\text{G}}_{{{\text{ij}}}}$ maxima in Fig. 5, reveal non-random pairing tendencies. There is general preference for cluster self-aggregation, maximum for ISRO ${\text{(A)}}$:${\text{G}}_{{{\text{AA}}}} > > {\text{G}}_{{{\text{CC}}}} > > {\text{G}}_{{{\text{BB}}}}$. Its defining rationale is the abundance ($n_{5}$) of pentagon faces available for pairing^24^ (Fig. 2d):${\text{A(n}}_{{5}} = {12)} > {\text{C(n}}_{{5}} = {10)} > {\text{B(n}}_{{5}} = {6})$. Strong ${\text{G}}_{{{\text{AA}}}}$ extending to ${0}{\text{.5 nm}}$ imply that ISRO self-aggregation extends over the whole MRO domain ${\text{(1 nm)}}$. Thus, ISRO networks can be concluded to constitute the backbone of glass structure. Inter-cluster separation between ISRO clusters (${\text{r}}_{{{\text{AA}}}} = {0}.{\text{25 nm}}$; Fig. 5a) is comparable with nearest neighbor bond-lengths (Table 3), which implies that two cluster centers are nearest neighbors of each other. This represents inter-penetrating ISRO clusters, conforming to efficiently packed networks^30^. In contrast, fewer pentagon faces for non-ISRO clusters limit their self-aggregation to nearest neighbors only (Fig. 5c). Thus, non-ISRO clusters form short networks ${(0}{\text{.7 nm)}}$. Separation of non-ISRO clusters ${\text{(r}}_{{{\text{CC}}}} = {0}.{\text{35 nm)}}$ is larger than nearest neighbor bond-length, which implies lesser inter-penetration of non-ISRO clusters. Thus, non-ISRO networks are less dense. On the other hand, the advantage of non-ISRO clusters is that they are non-preferential wrt choice of neighbors ${\text{(G}}_{{{\text{CA}}}} \approx {\text{G}}_{{{\text{CC}}}} {)}$. This allows their flexible accommodation as “glue” between ISRO networks.

**Figure 6:**
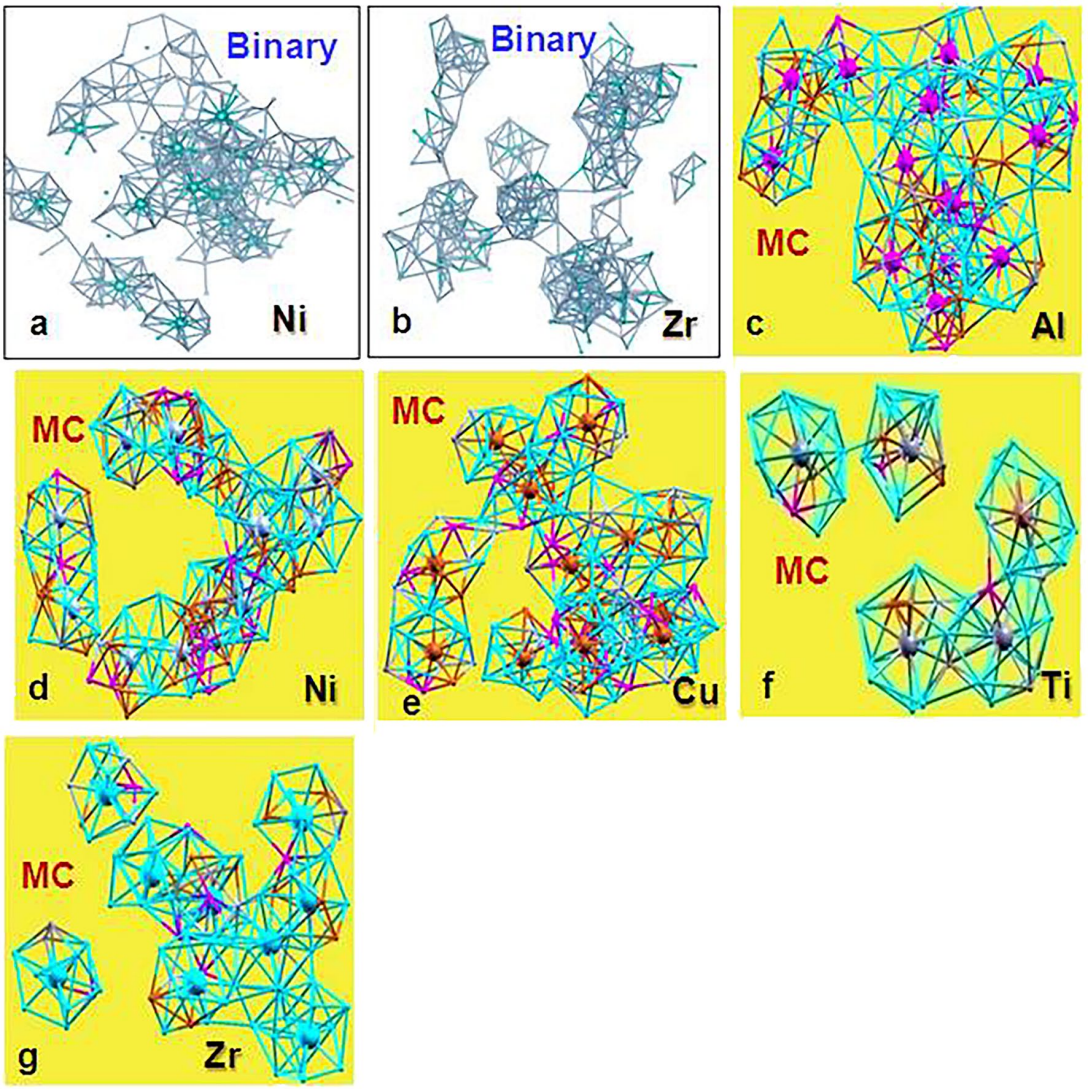
Arrangement of 12-fold coordinated polyhedra around different centers for (**a,b**) binary glass: (**a**) Ni, (**b**) Zr and (**c–g**) MC glass: (**c**) Al, (**d**) Ni, (**e**) Cu, (**f**) Ti, (**g**) Zr. Clusters are depicted using OVITO Basic v3.7.4 software (Ref. ^83^; https://www.ovito.org/windows-downloads/).

The formation of networks by ${\text{N}} = {12}$ clusters is depicted in Fig. 6. Center-specific clusters of ${\text{N}} = {12}$ were selected from FPMD ensemble and networks emanating from their connections are presented in Fig. 6a, b $(B)$ and Fig. 6c-g $(MC)$. For each glass, network size and density sharply decrease from smaller (e.g.${\text{Ni}}$) → bigger centers (${\text{Zr}}$), consistent with higher abundance of ${\text{(Al/Ni/Cu)}}$–centered clusters amongst ${\text{(N}} = {12)}$ cluster group.

### Fluctuation electron microscopy (FEM)

**Figure 7:**
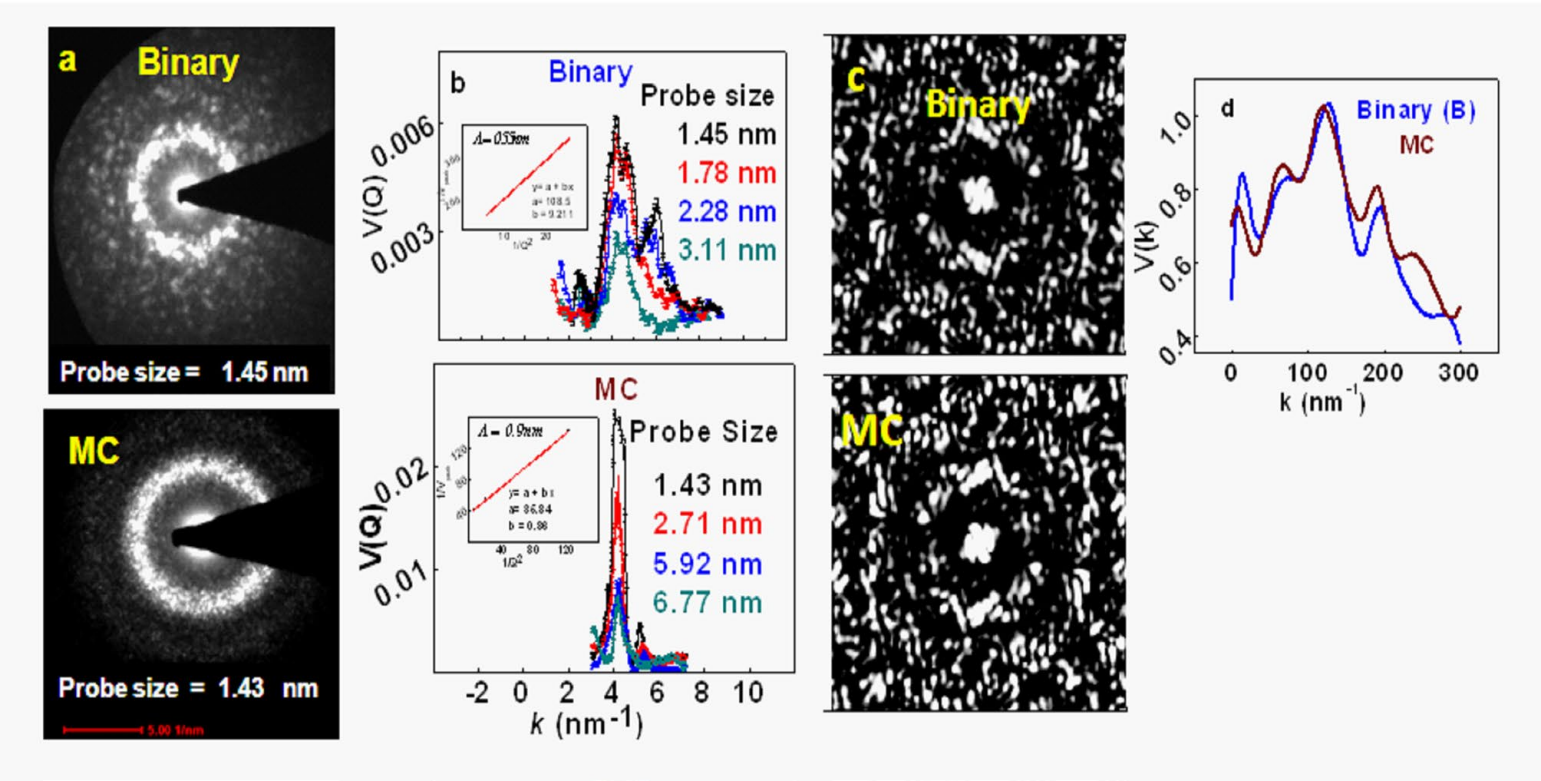
FEM results for binary (B) glass (upper panel) and multi-component (MC) glass (lower panel). (**a**) Nanobeam electron diffraction (NBED) patterns, corresponding to probe sizes 1.45 nm (B) and 1.43 nm (MC); (**b**) variance (V(k)) for 4 resolutions for each glass, calculated from respective NBED patterns. In respective insets, (1/V_peak_) vs. (1/Q^2^) is plotted. (V_peak_ = V(k) for the first peak; Q = inverse of probe size). These linear plots are fitted to derive respective MRO domain sizes; (**c**) Simulated FEM diffraction patterns, corresponding to FPMD model. Virtual probe size = 1.6 nm; (**d**) V(k) calculated from simulated diffraction patterns.

Variable Resolution FEM (VRFEM) was measured for $B$- and $MC$-glasses, with probe sizes ${(1}{\text{.45}} - {3}{\text{.11) nm}}$ and ${(1}{\text{.43}} - {6}{\text{.77) nm}}$ respectively. [Representative diffraction patterns for both glasses exhibit sharp ring (Fig. 7a), validating the existence of significant MRO. Variance ${\text{V(k)}}$ was determined from these patterns, for different probe sizes (Fig. 7b). Correlation between the first peak of ${\text{V(k)}}$[$= {\text{V}}_{{{\text{peak}}}}$] and probe size $({1 \mathord{\left/ {\vphantom {1 Q}} \right. \kern-0pt} Q})$ was generated [$1/V_{peak} ,Q$], shown in the respective insets of Fig. 7b. MRO domain sizes ${(}\Lambda {)}$ were derived from respective linear correlations^58,59^: [${{\Lambda (B)}} = {0}{\text{.55 nm}}$;${{\Lambda (MC)}} = {0}{\text{.9 nm}}$].

We next exploited FEM to probe atomic arrangements on MRO scale. Since FEM data cannot be directly inverted into unique atomic positions, we adopted an indirect method. Based on FPMD cluster arrangements, we simulated FEM spectra (Fig. 7c) with virtual probe size ${{\sim 1}}{\text{.6 nm}}$ ^55–57^. For each glass, simulated FEM patterns (Fig. 7c) and maxima positions of ${\text{V(k)}}$ (Fig. 7d) are reasonably comparable with experimental (Fig. 7a, b). This signifies that FPMD cluster arrangements do approximate the real network structure (fluctuation in simulated result is significantly higher than experimental since virtual probe size $= {1}{\text{.6 nm}}$ in all three dimensions while actual sample thickness $> > {1}{\text{.6 nm}}$. Such differences have been reported earlier^60–62^).

### Nanobeam electron diffraction (NBED)

**Figure 8:**
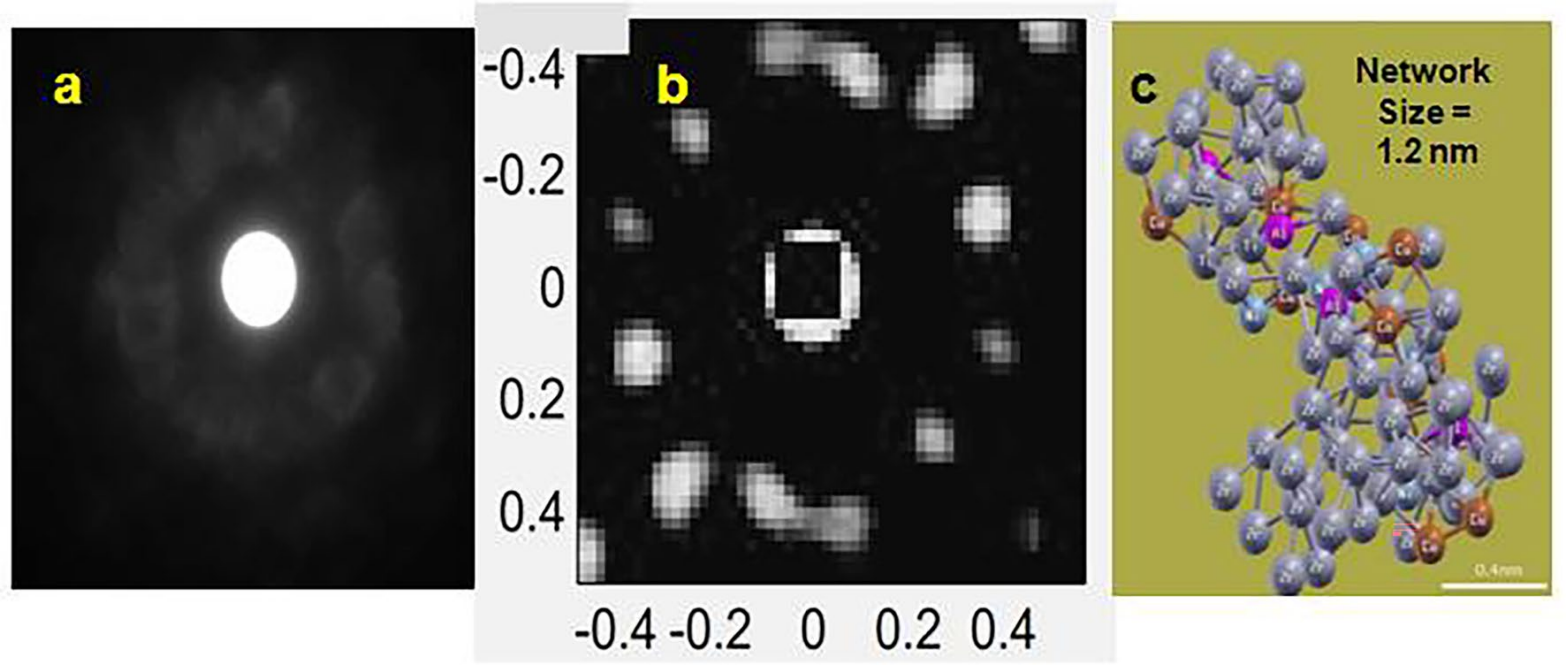
(**a**) Experimental and (**b**) simulated nano beam electron diffraction (NBED) spectra. (c) Cluster network, corresponding to simulated spectra, is depicted using OVITO Basic v3.7.4 software (Ref. ^83^; https://www.ovito.org/windows-downloads/). Intensity of the central spot has been masked (similar to beam stopper in experimental pattern) by square region, to enable visibility of the diffraction spots whose intensity is lower**.**

(MRO domain size of $B$-glass $( = {0}{\text{.55 nm)}}$ is shorter than NBED probe size $( = 1\;{\text{nm)}}$. Therefore, exercise of NBED technique is meaningless for $B$-glass). Experimental NBED pattern for $MC$-glass is displayed in Fig. 8a. Intensity of the diffraction spots for $MC$-glass (Fig. 8a) is lower than for individual clusters (Fig. 4a), consistent with decreasing order over medium range. Experimental pattern (Fig. 8a) matches closest with simulated diffraction pattern (Fig. 8b), corresponding to FPMD cluster network of Fig. 8c. This signifies that under suitable conditions of orientation, a set of clusters can be in definite arrangement over ${1}{\text{.2nm}}$ extent to generate well-defined diffraction patterns. This is the first reported detection of individual network and therefore, milestone in glass research.

### Free volume

#### Positron annihilation spectroscopy (PAS)

Results of PAS spectra (by some of the present authors in Ref. ^68^) are utilized in the present paper. $3$ distinct lifetime components are demonstrated for both glasses:

$$\begin{array}{*{20}c} {{{\tau }}_{{\text{1}}} {\text{(B)}} = {\text{0}}{\text{.2 ns (94}}{\text{.3\% )}}} \\ {{{\tau }}_{{\text{1}}} {\text{(MC)}} = {\text{0}}{\text{.19 ns (86}}{\text{.6\% )}}} \\ \end{array} ,\quad \begin{array}{*{20}c} {{{\tau }}_{{\text{2}}} {\text{(B)}} = {\text{0}}{\text{.566 ns (4}}{\text{.1\% )}}} \\ {{{\tau }}_{{\text{2}}} {\text{(MC)}} = {\text{0}}{\text{.277 ns (12}}{\text{.6\% )}}} \\ \end{array} ,\quad \begin{array}{*{20}c} {{{\tau }}_{{\text{3}}} {\text{(B)}} = {\text{1}}{\text{.8 ns (1}}{\text{.6\% )}}} \\ {{{\tau }}_{{\text{3}}} {\text{(MC)}} = {\text{1}}{\text{.8 ns (0}}{\text{.8\% )}}} \\ \end{array}$$

The longest lifetime (${\uptau }_{{3}}$) with low intensity is ignored since it corresponds to lifetime originating from surface of the sample. For each glass, the shortest ${{(\tau }}_{{1}} )$ and intermediate ${{(\tau }}_{{2}} )$ lifetime components represent defects within SRO domain and at the boundary of MRO domains (determined by FHREM in later section) respectively^65–68^. Relative intensity of these components ${\text{(I}}_{{1}} :I_{2} ) > > {1}$ is statistically consistent, since number of MRO domains is small fraction of the number of SRO domains. FV size within SRO domains is similar between the two glasses ${{[\tau }}_{{1}} (B)\sim \tau_{1} (MC)]$; on the other hand, FV size at the boundary of MRO domain is higher for $B$-glass ${{[\tau }}_{{2}} (B) = 2 \times \tau_{2} (MC)]$ due to less dense packing.

#### Filtered high resolution electron microscopy (FHREM)

**Figure 9:**
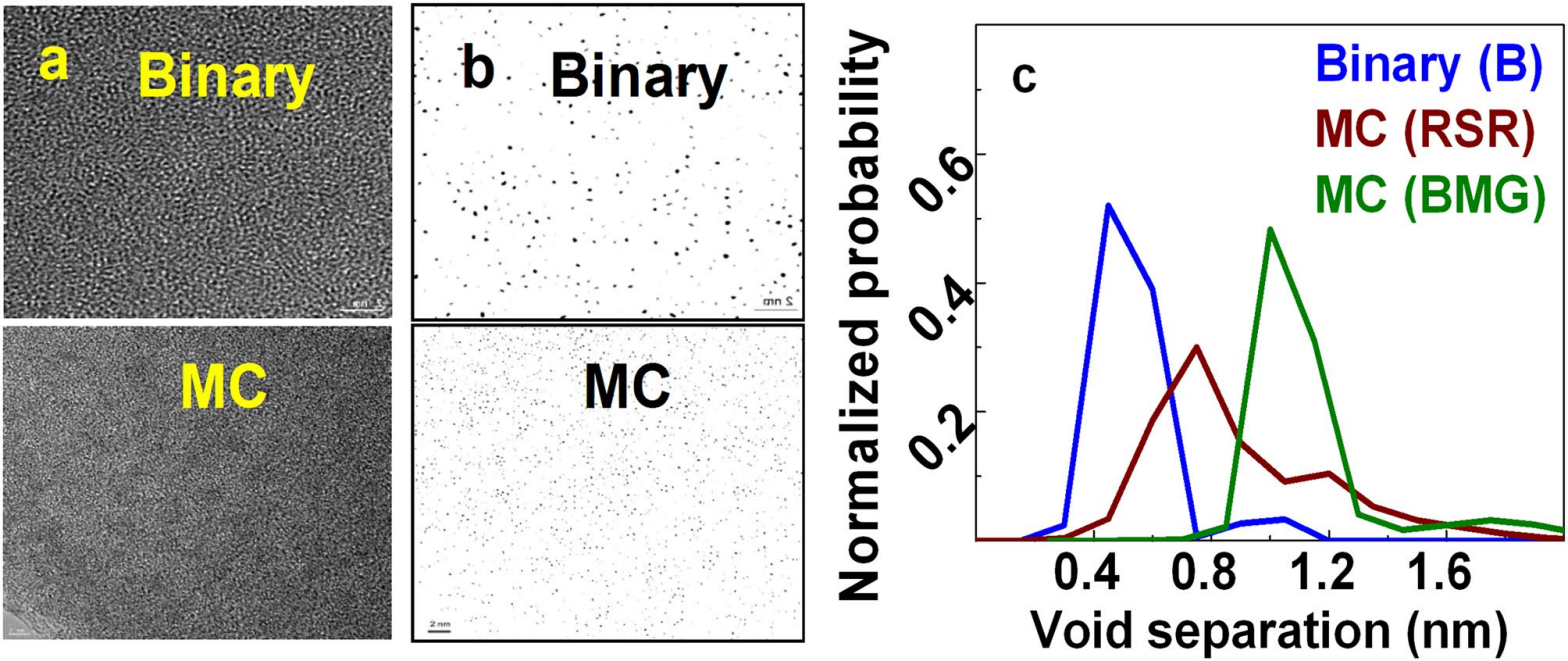
(**a**) High resolution electron microscopy (HREM) images of glasses; (**b**) Fourier filtered HREM (FHREM) images of the same, by applying annular mask. Voids are depicted as black dots; (**c**) distributions of inter-void separation for binary (B) and MC (RSR, BMG) glasses, derived from respective FHREM images (RSR = rapidly solidified ribbon; BMG = bulk metallic glass).

HREM images for $$B$$- glass and $$MC$$-glasses [RSR (rapidly solidified ribbon) & BMG] is presented in Fig. 9a; these images were filtered by applying annular mask to extract void distribution image, depicted as black dots in Fig. 9b (we coined the term “void” to refer to regions where FV is more concentrated^2^). Since voids of SRO domain are ubiquitous, no extra information can be gained out of them. We rather focused on the voids present between MRO domains. Since these voids are typically of $${0}{\text{.5 - 2 \, nm}}$$ size, only defects of this size range were filtered out by application of annular mask $$M(k)$$ ^67^. Therefore, FHREM images of Fig. 9a, b essentially represent voids between MRO domains. It is clear from these figures that void distribution is not homogeneous but consists of densely and loosely packed regions^75^. This generates (Gaussian) distribution of inter-void separation for each glass (Fig. 9c) (values for inter-void separation may be slightly underestimated since FHREM image is $${\text{2D}}$$ projection of $${\text{3D}}$$ sample. Nevertheless, this error can be considered secondary for thin samples as ours). Maxima of the distributions shift from $$0.5nm(B)$$$$\to 0.8{\text{ nm }}(RSR)$$$$\to 1.2{\text{ nm }}(BMG)$$ i.e. inter-void separation increases, consistent with densification from $$B \to MC(RSR) \to MC(BMG)$$. It is noted that inter-void separation values match with respective MRO domain sizes. This correlation could be interpreted to conclude that FV is present at MRO boundary.

## Discussions

Structure of metallic glass is unanimously predicted to be well-defined within “finite” degeneracy^38–41^. But its experimental realization is inherently difficult due to poor detection of amorphous structure. In this work, we undertook the formidable experimental challenge of resolving structural degeneracy to the best extent possible with the employment of ${7}$ contemporary multi-scale (SRO, MRO, FV) techniques on the *same* glass. The strategy was exercised on two glasses [${\text{Zr}}_{{{67}}} {\text{Ni}}_{33} (B)$,${\text{Zr}}_{{{52}}} {\text{Ti}}_{{6}} {\text{Al}}_{{{10}}} {\text{Cu}}_{{{18}}} {\text{Ni}}_{14} (MC)$] to ensure that our conclusions are not fortuitous but commensurate with GFA of glasses. The structural basis of these glasses is formed of ${\text{3(B)}} \to {\text{15(MC)}}$ partial pair correlations. Degeneracy subsequently multiplies in successive stages, starting from distribution of bond parameters for each pair → several combinations of these pairs into a range of cluster configurations → variable inter-cluster correlations leading to variable MRO → correlations between MRO → accommodations of FV. These cumulatively build a plethora of possible configurations. We critically evaluate the success of our experimental strategy in resolving this degeneracy. We clarify that direct reconstruction of structure from experimental data is non-unique for most of our techniques, which necessitated a reference model for experimental data-fitting. Reference structural model was generated by FPMD simulations for $(250 - 686)$-atom ensembles at $10^{13} {\text{K/s}}$. FPMD results are reportedly robust against variations of ensemble size $( \pm 2\% )$. Robustness relative to ensemble size is justified by the exercise of exact potentials in FPMD (without explicit assumption, unlike classical MD). These demonstrate the practical utility of smaller ensemble sizes in FPMD simulations.

### SRO

The ultimate target for SRO information comprises (coordination, composition, bond-length, distortion and symmetry) parameters of *individual* clusters. These aspects have been addressed in our work by global (XRD, XAFS, FEM) and local (3D-APT, ABED) tools, within their respective limitations. XAFS resolved the degeneracy between ($Ni,Cu,Zr$)-centered cluster groups wrt (site-averaged) bond-length (and cluster distortion) parameters. These results are unique and consistent with FPMD model, within $\pm {0}{\text{.1 {\AA}}}$. Although XAFS is incapable of resolving individual clusters, these results practically represent individual clusters due to small inter-cluster variance (DWF). Thus, bond-length (and cluster distortion) information for whole ensemble is practically complete from XAFS. On the other hand, coordination (and composition) information is inaccessible from XAFS since its interpretation for glass is intrinsically ambiguous^20^. This gap was partly filled by 3D-APT, through direct conversion of APT spatial atomic distribution into partial pair coordination. Although absolute values of APT coordination are meaningless (due to atomic loss during experiments), they uniquely reproduced fractional composition of FPMD clusters within $\pm {\text{5\% }}$. Cluster coordination and symmetry information could not be solved independently from any technique but indirectly validated through parallel reproduction of FEM, XRD and ($\times {3}$) XAFS spectra with FPMD atomic coordinates. Such validation would appear non-unique, if each technique was considered independently. The strength of our work lies in that this ambiguity is considerably reduced through mutual corroboration by multiple techniques. These validations warrant that FPMD clusters are at least realistic. The most direct access to *individual* clusters is through ABED, although the probability of detection is low ($\le 2$ clusters). Experimental ABED patterns were reproduced by specific FPMD clusters (out of $\ge 500$-atom ensemble). Hence, information of these individual clusters can be concluded to be reasonably unique. Thus, combination of (XRD, XAFS, APT, FEM, ABED) generated comprehensive information of clusters—(bond-length, distortion, fractional composition) uniquely and (coordination, symmetry) indirectly. This narrowed down degeneracy of SRO toward finite (FPMD) model with reasonable certainty (vis-à-vis previous efforts).

### MRO

The ultimate target for MRO information comprises detection of *individual* networks and identification of cluster-pairing scheme. MRO analysis is formidable (relative to SRO) due to decreased order in this domain and corresponding detection limitations. There is no direct experimental access to MRO information (unlike XAFS/3D-APT), except for MRO domain size $\left( {\Lambda } \right)$ ^58,59^ with VRFEM technique. Solution for ${\Lambda }$ from these techniques is unique and model-independent and matched with FPMD domain size for $(B,MC)$ glasses, within $\pm {0}{\text{.15 nm}}$. In this work, we undertook the additional (formidable) challenge of retrieving cluster arrangement information with global (FEM) and local (NBED) tools, supported by FPMD modeling (NBED was not exercised for $B$- glass since its MRO domain size is shorter than probe size). (a) Reproduction of FEM spectra with FPMD atomic coordinates (for both glasses) and (b) NBED pattern (for $MC$-glass) with *one* particular network of ${1}{\text{.2 nm}}$ extent (out of $\ge 500$-atom ensemble) mark significant progress towards unique solution. These experimental validations corroborate the inherent non-random cluster-pairing tendencies of FPMD model (e.g. preferential pairing of ISRO clusters). In particular, NBED detection of robust ISRO network is the first direct evidence of self-aggregation tendency of ISRO clusters. Although various types of polyhedral packing are suggested by other workers^2,5,31,37^, we did not find unambiguous evidence for any of these.

### FV

A complete understanding of the structure and properties of glass does not emerge without accounting for the intrinsic open space (FV) distribution^32–35^. Existence of FV does not contradict the fact that metallic glasses are strong materials. We clarify that the word “void” in our FV description does not imply the existence of pores (as for porous material) but specific regions of glass structure where free volume (FV) is localized. PAS was traditionally the only access to FV content information^64–66^. Recently-developed FHREM imaging technique^67^ directly reveals precise inter-void separation information. However, these information pieces independently do not enhance structural understanding of glass unless integrated with SRO and MRO information. We undertook a novel approach to determine the location of FV, by synergizing inter-void separation (FHREM; Fig. 9c) and MRO domain size (NBED/FEM) information. Besides $$B$$- and $$MC$$$$(RSR)$$-glasses, FHREM of reference $$MC$$ $$(BMG)$$ was measured for systematic conclusion. For each glass, inter-void separations matched with MRO domain size $$\left( {\Lambda } \right)$$. This unambiguously pinpointed the presence of FV at boundary of MRO domain. Presence of FV at MRO boundary provides the first experimental representation of intermediate region between MRO domains. Atoms of such low-density intermediate region do not form well-defined clusters but act as “gel” atoms connecting MRO domains^76^. Such image was missing from earlier studies.

We remark that atomistic simulation of FV was not indispensable for this work, since FV information was directly and unambiguously determined by (PAS, FHREM) techniques.

### SRO–MRO–FV correlation

In crystalline materials, a motif repeats in a specific way to generate long-range structure so that SRO → MRO → LRO link is uniquely defined and readily detectable with diffraction. Experimental reconstruction of hierarchy for glasses is intrinsically non-trivial, due to (i) poor detection and (ii) degeneracy of solution. Nonetheless, our multi-scale experimental approach enabled the reconstruction of *one* complete hierarchical subset for $MC$-glass, by integrating the results from (NBED, PAS, FHREM) techniques. The first leg of the hierarchy viz. SRO → MRO was reconstructed by NBED (with reasonable certainty), for a FPMD network subset of face-sharing clusters (Fig. 8). Inhomogeneous spatial distribution of FV within network was independently determined with PAS. The next leg of hierarchy was completed with the detection of FV (or region of low atomic density) at MRO boundary, with FHREM. This intermediate region serves as the connecting link between network domains: MRO → FV → Bulk. The two legs of hierarchy jointly reconstructed (at least) *one* subset of the complete structure: [SRO → MRO → FV → Bulk], which was never attempted in earlier works. As the probability of network detection with NBED is rather low and unpredictable (subject to the appropriate orientation for diffraction), we remark that the number of hierarchical subsets that can be reconstructed is a matter of chance. The importance of our exercise lies in demonstrating the pathway for achieving the same.

### Comparison of glasses

Our work demonstrates that complication of experimental analysis of structure does not necessarily multiply with the number of atomic components of the glass e.g. from $B$ → $MC$ glass. In fact (for example), larger extent of information was retrieved for MRO of $MC$-glass (vis-à-vis $B$), through the detection of larger number of clusters and extended network. This is rational, since most of the experimental techniques (FEM, XRD, ABED/NBED, PAS, FHREM) are insensitive to the number of atomic components. The element-sensitive techniques (XAFS, 3D-ATP) also could handle $(B,MC)$ glasses with comparable flexibility. XAFS for $MC$-glass was practically simplified as: (i) $(Al,Ti)$-site information was inaccessible due to inadequate photon flux at these edges. This limited XAFS analysis to $(Ni,Cu,Zr)$-centers [vis-à-vis $(Ni,Zr)$ for $B$-glass]; (ii) $(Ni,Cu)$ neighbors are indistinguishable, so that compositional analysis involved $(Al,Ni,Zr)$ atoms [vis-à-vis $(Ni,Zr)$ for $B$-glass]. [$Ti$ is statistically excluded due to dilute content.] Thus, XAFS analysis for $MC$-glass is essentially reduced to solving [*one extra* site $\times$ *one extra* pair correlation per site] *i.e.* $5$ *extra* pair correlations, relative to $B$-glass. Since these correlations are well-resolved *wrt* atomic contrast and bond-lengths, they were easily solved (at par with $B$-glass).

The structure of $(B,MC)$ glasses is commensurate with their relative GFA $(MC > B)$:

(a) Strong $Ni - Zr$ chemical interaction of $B$-glass plays a key role in destabilizing ISRO and driving toward crystalline $(NiZr_{2} )$-like clusters. Poor ISRO content and presence of $(NiZr_{2} )$-like clusters are consistent with poor GFA of $B$-glass. In contrast, competition of various chemical interactions in $MC$-glass prevents the precipitation of any particular crystalline phase. ISRO is the dominant cluster symmetry group for $MC$-glass.

(b) Disparity of SRO generates discrete MRO for $(B,MC)$ -glasses. Shortage of ISRO clusters fails to support extended networks in $B$-glass. In contrast, ISRO networks of $MC$-glass extend up to $0.9nm$ on average (vis-à-vis $0.55{\text{nm}}$ for binary glass). Large MRO domains of $MC$-glass indicates the presence of large regions that have order different from crystalline phase. This alternatively represents highly disordered structure, consistent with higher GFA^57^.

## Conclusions

We accomplished the first precise experimental reconstruction of a subset of structural hierarchy (SRO → MRO → FV → bulk structure) for any metallic glass, with the examples of ${\text{Zr}}_{{{67}}} {\text{Ni}}_{{{33}}}$ and ${\text{Zr}}_{{{52}}} {\text{Ti}}_{{6}} {\text{Al}}_{{{10}}} {\text{Cu}}_{{{18}}} {\text{Ni}}_{{{14}}}$ glasses. Employment of $7$ multi-scale techniques (encompassing all domains) is the key to this success. This strategy complemented mutual limitations of individual techniques, corroborated common results and established correlation between (SRO, MRO, FV) domains, to present the most complete and unambiguous structural characterization for any glass to date. In SRO domain, site-resolved cluster size, composition and symmetry parameters were precisely defined with (XRD, XAFS, ABED, 3D-APT) techniques. Non-random linkage of clusters into the formation of MRO domain of size $\sim 0.55{\text{nm}}(B) - 0.9{\text{nm}}(MC)$ was established by (FEM, NBED) techniques. These experimental results narrowed down the degeneracy of structural solution toward FPMD model. This match is not fortuitous but consistent with relative GFA of the glasses. MRO domains get eventually broken beyond $\sim 0.9{\text{nm}}$ due to lack of long range order, which confirms that similarity with crystalline structure is primarily limited to SRO and ends within MRO length-scale. In the case of glass, this necessitates the presence of free volume, which were precisely characterized for content and distribution by (PAS, FHREM) techniques. As inter-void separation matched with respective MRO domain sizes for both glasses, this correlation was judiciously exploited to pinpoint the location of FV at MRO boundary. Thus, FV or “gel” atoms act as the link between MRO domains (MRO → FV → bulk structure)—a picture that is experimentally unravelled for the first time. Cumulatively, the bottom-up structural hierarchy of metallic glass (SRO → MRO → FV → bulk structure) is constructed and visualized like never before. Our work should direct and inspire future studies towards finite structural solution for metallic glass.

## Methods

### First-principles molecular dynamics (FPMD)

FPMD simulations are performed with finite temperature density functional theory as implemented in the VASP code^77^. These simulations employ projector augmented wave (PAW) potentials (as supplied with code) and PBE-GGA flavor of exchange–correlation. Plane wave basis set is constructed with energy cutoff ${\text{500eV;}}$ Brillouin zone integrations were performed using ${\Gamma }$-point. Simulations were performed on $500$-atom and ${250 - 686}$-atom cubic supercells for $B$- and $MC$-glasses respectively, with periodic boundary conditions. Initial configuration is generated by randomly distributing atoms inside the simulation box at experimental density. The simulations are carried out in a canonical ensemble (*NVT*) with Nose´ thermostat for temperature control and the equations of motion are solved with ${\text{2 fs}}$ time step. First, the system is melted at ${\text{3000 K}}$, followed by ${\text{5 ps}}$ equilibration period. The system is next instantly quenched to ${\text{500 K}}$, followed by ${\text{50 ps}}$ equilibration period. Data for the last ${\text{10 ps}}$ is used for structural analysis. Voronoi tessellation method^2^ was used to identify cluster geometries (each Voronoi polyhedron is defined by indices $\left\langle {{\text{n}}_{{3}} {\text{,n}}_{{4}} {\text{,n}}_{{5}} {,}..} \right\rangle$, where ${\text{n}}_{{\text{i}}}$ denotes its number of *i*-edged faces) (for example,${\text{n}}_{{5}}$ denotes number of pentagonal faces).

### Sample preparation

Zr_67_Ni_33_ and Zr_52_Ti_6_Al_10_Cu_18_Ni_14_ alloys were produced by vacuum arc melting of pure metals in right proportions. Representative rapidly solidified ribbon (RSR) of thickness $= 20 - 30{\mu m}$ and width = 5 mm (Fig. S1a) were produced from these alloys by melt spinning technique^78^. Amorphous phase of the ribbon was confirmed by absence of sharp crystalline peaks in XRD (Fig. S1b) and indistinguishable lattice fringes in HREM image (Fig. S1c) (bulk metallic glass (BMG) counterpart of Zr_52_Ti_6_Al_10_Cu_18_Ni_14_ was synthesized at slower cooling rate, to be used for FHREM measurement).

### X-ray diffraction

X-ray diffraction pattern of the glass ribbons was obtained on image plate, at BL-11 beamline (Indus-2) with synchrotron X-ray source ($\lambda = {0}{\text{.407 {\AA}}}$) in transmission mode. XRD image was converted to the standard X-ray diffraction pattern $(I,2\theta )$ with FIT2D open source software. XRD patterns (Fig. S1b) displayed broad peaks, characteristic of amorphous structure. Radial distribution function $G(R)$ was calculated from XRD data with PDFgetX3 open source software^79^.

### X-ray absorption fine structure (XAFS)

For $$B$$-glass, structure around $${\text{Zr}}$$-site was obtained by tuning the incident X-ray energy to $$Zr$$$$K$$-edge ($${\text{Ni}}$$$$K$$-edge dataset varied rapidly within the course of single scan (< 20 min), as the glass started annealing. This is due to heat absorption from incident X-rays, facilitated by large absorption cross-section at $${\text{Ni}}$$$$K$$-edge. This is proof that the structure of $$B$$-glass is close to crystalline phase). For $$MC$$-glass, $${\text{(Ni,Cu,Zr)}}$$ site-resolved structures were obtained by selectively tuning the incident X-ray energy to $${\text{(Ni,Cu,Zr)}}$$$$K$$-edges respectively ($${\text{(Al,Ti)}}$$$$K$$-edge XAFS were not measured due to unavailibility of adequate flux at these energies). XAFS spectra on as-cast ribbon samples were recorded in transmission mode at MRCAT-ID, Advanced Photon Source (USA)^80^. Si (111) monochromator in conjunction with harmonic rejection mirror was used to filter out the required energy. Argon and Krypton filled ionization chambers were used to monitor the incident and transmitted X-ray intensities, respectively. XAFS data were processed using ATHENA software^81^. Oscillations $$\chi (E)$$ are extracted following background subtraction and normalization. The energy scale is converted to wave number scale *k* given by $$k = \left( {\frac{{2m(E - E_{0} )}}{{\hbar^{2} }}} \right)^{1/2}$$ , where *m* = electron mass and *E*_0_ = edge energy of the relevant absorption edge. $$\chi (k)$$ were Fourier-transformed over $$\Delta {\text{k}} = {2}{\text{.5 - 10 {\AA}}}^{{ - 1}}$$ into real space χ(*R*) for fitting over *R*-range ($$\Delta {\text{R}} \approx {1}{\text{.5 - 3}}{\text{.2 {\AA}}}$$). Theoretical scattering amplitudes and phases were generated by FEFF6 program and used in fitting program FEFFIT^82^. *R*-factor was considered as estimate of fit quality. Good *R*-factor (< 0.01) was obtained for all our fits.

### Angstrom/nano beam electron diffraction (ABED/NBED)

ABED was performed using aberration-corrected transmission electron microscope (FEI-TITAN TEM). ABED patterns were acquired from the thinnest region (edge of the hole) of the metallic glass samples in STEM mode with ${0}{\text{.5 nm}}$ size electron probe, which was generated using ${5 }\mu {\text{m}}$ condenser apertures. Thickness of the sample at the thin region was estimated to be $< {\text{10 nm}}$ by electron energy loss spectrometer. Nanobeam electron diffraction (NBED) is an extension of ABED, with coherent electron beam probe size increasing to ${\text{1 nm}}$, which enables the detection of networks. For simulation of ABED/ NBED patterns, intensity of electron diffraction $(I(\mathop {\text{Q}}\limits^{ \to } ))$ from cluster was calculated using the relation: ${\text{I(}}\mathop {\text{Q)}}\limits^{ \to } = {\text{N}}\left| {{\text{f(}}\mathop {\text{Q}}\limits^{ \to } {)}} \right|^{{2}} \left( {{1} + \sum\nolimits_{{{\text{m}} \ne {\text{n}}}} {{\text{exp}}^{{{ - 2}\pi \mathop {{\text{Q}}{.}\mathop {{\text{r}}_{{{\text{nm}}}} }\limits^{ \to } }\limits^{ \to } }} } } \right)$ ^46^ ($\mathop {\text{Q}}\limits^{ \to }$ = scattering vector, ${\text{f(}}\mathop {\text{Q)}}\limits^{ \to } =$ electron scattering factor of individual atoms;$\mathop {\text{r}}\limits^{ \to }_{{{\text{nm}}}} =$ distance between correlated ${\text{(n,m)th}}$ atoms in clusters). Since cluster orientation in experimental sample can be random, all possible patterns (corresponding to different zone axes) for all possible FPMD cluster symmetries were simulated. Entire reciprocal space in ${\text{3D}}$ was evaluated in grid of ${0}{\text{.01 {\AA}}}^{ - 1}$ for reciprocal length (${0}{\text{.5 {\AA}}}^{ - 1}$). Parallel ${\text{c}}^{ + + }$ code and visualization programs were respectively developed for simulation and extraction of ${\text{2D}}$ patterns from ${\text{3D}}$ computed diffraction data. Clusters, corresponding to simulated pattern, were depicted using OVITO Basic v3.7.4 atomistic visualization software^83^.

### Fluctuation electron microscopy (FEM)

FEM was measured in Variable resolution (VRFEM) mode with varying probe size (${1}{\text{.4}} - {6}.7{\text{nm}}$)^59^. The smallest probe size (${1}{\text{.4 nm}}$) is expected to cover ${6} - {7}$ clusters, as suggested by Fig. 6. Increasing probe size covers more cluster units with decreasing correlation until diffraction pattern is reduced to amorphous ring. This transition length scale is identified as MRO domain size (${\Lambda }$), beyond which cluster correlation vanishes. Diffraction patterns were acquired with ${10} \times {10}$ grid positions in ${100} \times {10}0{\text{nm}}^{2}$ area. Samples for TEM were prepared by window thinning technique using electrolyte comprising ${\text{80\% }}$ methanol,${\text{20\% }}$ perchloric acid by volume. Temperature of electrolyte was maintained at ${\text{T}} < {\text{220 K}}$. Hwang et al*.* method^60^ was adopted to eliminate thickness effect on variance with high angle annular dark field (HAADF) image intensity as thickness reference.

Strength of spatial fluctuations is quantified by normalized variance (${\text{V}}$) of diffracted intensity: ${\text{V(K,Q)}} = \frac{{\left\langle {{\text{I}}^{{2}} \left( {\text{r,k,Q}} \right)} \right\rangle }}{{\left\langle {{\text{I}}\left( {\text{r,k,Q}} \right)} \right\rangle^{{2}} }} - {1}$, where ${\text{I(r,k,Q)}}$ is the image intensity as function of position ${\text{(r)}}$ in the image, scattering vector ${\text{(k)}}$ and objective aperture size (${\text{Q}} \propto \tfrac{{1}}{{\text{D}}};{\text{D}} =$ probe size). For quantitative determination of domain size, experimental ${\text{V(k,Q)}}$ are constructed in Fig. 7b. $\left( {\frac{{1}}{{\text{V}}}{,}\frac{{1}}{{{\text{Q}}^{{2}} }}} \right)$ follows linear equation: $\frac{{1}}{{\text{V(k,Q)}}} = {\text{c}} + \frac{{\text{m}}}{{{\text{Q}}^{{2}} }}$ (inset of Fig. 7b), from which offset ${\text{(c)}}$ and slope ${\text{(m)}}$ were derived;${\text{(c,m)}}$ results are inserted into equation $\left( {{\Lambda } = \frac{{1}}{{{{2\pi }}}}\sqrt {\frac{{\text{c}}}{{\text{m}}}} } \right)$ to derive ${\Lambda }$.

For validating atomic arrangements on MRO length scale, we adopted indirect method of simulating FEM pattern and comparing with experimental pattern. We simulated FEM patterns corresponding to FPMD-generated atomic coordinates within cubic super cell of size ${\text{a}} = {1}{\text{.6nm}}$. Diffraction patterns were generated (Fig. 7c) by extracting ${\text{2D}}$ slice in ${\text{3D}}$ reciprocal volume and variance ${\text{V(k)}}$ was derived (Fig. 7d) from ${\text{I(r,k,Q)}}$ pattern.

### Atom probe tomography (3D-APT)

Samples for APFIM were prepared using dual-beam FEI Helios Focused Ion Beam (FIB) microscope, in combination with Omniprobe micromanipulator. FIM was performed at ${\text{30 K}}$ under neon pressure of ${10}^{{ - {5}}}$ Torr using Cameca FlexTAP instrument. APT was performed utilizing both electrical and laser-pulsing modes, employing LEAP tomography (LEAP 5000 XS and XR, Cameca). Specimen temperature was maintained at ${\text{30 \, K}}$. LEAP tomographic data were analyzed employing the program IVAS (Cameca). Additional details are provided in Ref. ^84^. Spatial coordinates ${(} \pm {0}{\text{.2 \, nm)}}$ and elemental identities of ${{\sim 10}}^{{7}}$ atoms were generated by 3D-APT for selected volume. Atoms within sub-volume ${(10} \times {10} \times {\text{8) nm}}^{{3}}$ were selected for analysis and Python-based code was developed for calculating partial pair coordination ${\text{(N}}_{{{\text{ij}}}} {)}$ around each atom.

### Positron annihilation spectroscopy (PAS)

Positron life time spectra were obtained by PAS (over $\varphi = 6mm$). Positron life time spectra (time ${(}\tau {)}$ versus counts ${\text{(N)}}$) was fitted with the equation below to obtain lifetime components ${{(\tau }}_{{\text{i}}} {)}$ with their respective frequencies ${\text{(I}}_{{\text{i}}} {)}$: ${\text{N(t)}} = \sum\limits_{{{\text{i}} = {1}}}^{{{\text{k}} + {1}}} {\frac{{{\text{I}}_{{\text{i}}} }}{{{\uptau }_{{\text{i}}} }}} {\text{exp(}} - \tfrac{{\text{t}}}{{{\uptau }_{{\text{i}}} }}{)}$. Positron annihilation lifetime was measured on the samples in sandwich configuration, using fast–fast coincidence spectrometer with ${\text{Na22}}$ positron source. Lifetime spectra (~ 106 counts) were analyzed with POSITRONFIT code^85^.

### Filtered high resolution electron microscopy (FHREM)

Details of this technique are provided in Ref. ^67^. All high-resolution images were recorded at nearly Scherzer defocus value. (TEM, HREM) were examined under JEOL 2000FX and TITAN microscopes respectively. Projected density fluctuations on the scale ${0}{\text{.5}} - {\text{2 \, nm}}$ were studied under weak phase-object approximation, which is valid for larger thicknesses in amorphous material. The above length scales were selected by the application of annular mask $M(k)$ that filters corresponding spatial frequencies. The net image intensity can be expressed as the convolution: $I_{f} (x,y) = 2\sigma \varphi (x,y)*FT\left[ {CTF \times M(k)} \right]$, where $\sigma =$ interaction constant;$\varphi (x,y) =$ projected potential, which is directly proportional to atomic density;${\text{CTF}} =$ contrast transfer function. Approximating $FT\left[ {CTF \times M(k)} \right]$ with sharp Gaussian-like peak, the equation reduces to $I_{f} (x,y) = 2b\sigma \varphi (x,y)$ [$b =$ constant]. Thus, intensity of the filtered image directly maps the atomic density fluctuation [$\propto {{\varphi (x,y)}}$]. To identify actual defects, threshold filter with annular mask ${(0}{\text{.5}} - {1}{\text{.5 \, nm}}^{{ - {1}}} {)}$ was set to pass regions which exceed the mean brightness by at least three standard deviations. Contrast in the image was inverted so that the bright features appear dark.

### Supplementary Information

Supplementary Information.

## Data Availability

The datasets used and/or analysed during the current study available from the corresponding author on reasonable request.
