# Supplementary material for: Comprehensive characterization of the structure of Zr-based metallic glasses
Source: Sci Rep. 2024 Feb 28;14:4911. doi: 10.1038/s41598-024-53509-y (PMC10902397; doi:10.1038/s41598-024-53509-y)
Supplement: Supplementary file 1 — Supplementary Information. [file 41598_2024_53509_MOESM1_ESM.doc]

**Comprehensive Characterization of the structure of Zr-based metallic glasses**

Debdutta Lahiri^1^*, K. V. Mani Krishna ^2^, Ashok K. Verma^1*^, P. Modak^1^, B. Vishwanadh ^2^, Soma Chattopadhyay^3^, Tomohiro Shibata^4^, S. K. Sharma^5^, Sudip Kumar Sarkar^2^, Peter H. Clifton^6^, A. Biswas^2^, Nandini Garg^1^ & G. K. Dey^7^

**Supplementary Material S1**

**Description of Techniques**

**X-ray Diffraction** is the standard technique for characterization of crystal structure. In the case of amorphous materials (as ours), it displays broad hump which is converted to generate radial distribution function for the system.

**X-ray Absorption Fine Structure (XAFS)** is an oscillatory feature above the x-ray absorption edge ($\text{E}_{0}$) of a constituent atom of the sample, resulting from the interference between outgoing photoelectron wave and the backscattered wave from the surrounding atoms^43^. By analyzing the period and amplitude of these oscillations, detailed structural information of the neighboring atoms viz. atomic species, coordination (*N*), bond-lengths (*R*) and mean square displacement ($\text{N,R,σ}^{2}$) *i.e*. site-resolved SRO can be obtained. The principle of XAFS is not based on long-range-order, which makes it unique probe for amorphous systems.

**Atom probe tomography (3D-ATP)** provides atomic-resolution imaging of the specimen^47-50^. Spatially resolved identity of the atoms is determined by time-of-flight measurement on position-sensitive detector. 3D-APT used in this work provides the spatial coordinates and elemental identities of atoms so that their distribution can be reconstructed in three-dimensions.

**Angstrom Beam Electron Diffraction (ABED)** is based on spherical aberration-corrected scanning transmission electron microscope that employs focused electron beam of size ~0.5 nm, comparable with the size of clusters. Thus, it is direct probe of actual arrangement of atoms in *individual* clusters^54^. The beam size can be increased from sub-nano → nano size (NBED) to cover more cluster units and detect MRO.

**Fluctuation Electron Microscopy** **(FEM)** is hybrid diffraction-imaging technique that measures spatial fluctuations in electron diffraction intensity, which is sensitive to higher-order (three and four-body) atomic correlations^58-59^. Fluctuations reveal the size, density and atomic arrangements of nanometer‐scale order (MRO).

**Positron Annihilation Spectroscopy (PAS)** is a powerful technique for characterizing sub-atomic open volume in glass (or any material)^64-66^, based on the interaction of positrons with electrons of the material and self-annihilation to produce gamma ray. Annihilation probability or conversely positron lifetime is essentially dictated by electron density; longer positron lifetime represents depleted electron density at the trap site or conversely larger size of the open volume.

**Filtered High Resolution Electron Microscopy (FHREM)** is adaptation of TEM for imaging defects in amorphous material, in which regions of atomic density significantly lower than the average are identified by suitable Fourier filtering and image thresholding techniques^67^.

**Figure S1**
